# A Heterobimetallic Anionic 3,6-Connected 2D Coordination Polymer Based on Nitranilate as Ligand

**DOI:** 10.3390/polym8030089

**Published:** 2016-03-16

**Authors:** Samia Benmansour, Carlos J. Gómez-García

**Affiliations:** Instituto de Ciencia Molecular (ICMol), Universidad de Valencia, C/Catedrático José Beltrán 2, 46980 Paterna, Valencia, Spain

**Keywords:** anilato ligands, heterometallic, coordination polymers, magnetic properties, high spin Fe(III) complex

## Abstract

In order to synthesize new coordination polymers with original architectures and interesting magnetic properties, we used the nitranilate ligand (C_6_O_4_(NO_2_)_2_^2−^ = C_6_N_2_O_8_^2−^), derived from the dianionic ligand dhbq^2−^ (2,5-dihydroxy-1,4-benzoquinone = H_2_C_6_O_4_^2−^). The use of this bis-bidentate bridging ligand led to [(DAMS)_2_{FeNa(C_6_N_2_O_8_)_3_}·CH_3_CN]*_n_* (1) (DAMS^+^ = C_16_H_17_N_2_^+^ = 4-[4-(dimethylamino)-α-styryl]-1-methylpyridinium), a 2D heterometallic coordination polymer presenting an unprecedented structure for any anilato-based compound. This structural type is a 3,6-connected 2D coordination polymer derived from the well-known honeycomb hexagonal structure, where Fe(III) ions alternate with Na^+^ dimers (as Na_2_O_12_ units) in the vertices of the hexagons and with an additional [Fe(C_6_N_2_O_8_)_3_]^3−^ anion located in the center of the hexagons connecting the three Na^+^ dimers. The magnetic properties of compound 1 show the presence of paramagnetic isolated high spin Fe(III) complexes with a zero field splitting, |D| = 8.5 cm^−1^.

## 1. Introduction

Coordination polymers, including its subgroup of metal organic frameworks (MOFs) or porous coordination polymers, represent a very active research area mainly due to the huge structural diversity [1,2,3,4,5,6] of these solids and the many interesting and varied properties that they may present. Thus, properties such as porosity [7], gas adsorption [8], ionic exchange [9], catalysis [10], energy production [11], gas separation [12], electrical [13] and proton conductivities [14], luminescence [15,16], ferroelectricity [17], magnetism [18], and non-linear optics [19] have been reported in coordination polymers. In some cases, the materials are multifunctional and show two or more of these properties simultaneously [20]. A wise choice of the precursor building blocks (tectons) and their interactions (synthons) [21] may lead to the formation of many different structures and topologies [22]. Two important steps forward are the so-called secondary building units (SBU) approach [23] that has resulted in coordination polymers and MOFs with controlled structures and porosities [23,24,25] and the complex-as-ligand approach [26,27,28], where a pre-formed complex containing additional free coordinating atoms can play the role of a ligand to coordinate with other metal ions to form homo- or heterometallic coordination polymers.

**Scheme 1:**
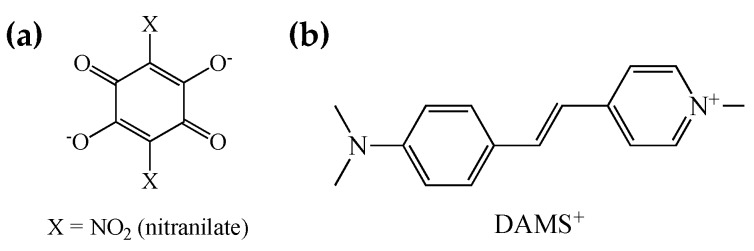
Structures of (**a**) nitranilate ligand and (**b**) DAMS^+^ cation.

In the last two years, we have been using anilato derivatives of the type C_6_O_4_X_2_^2−^ (X = H, Cl, Br, I, and NO_2_, Scheme 1) to prepare several new families of heterometallic coordination polymers including: (i) hexagonal honeycomb layers exhibiting porosity and chirality where the magnetic ordering temperature can be easily tuned by changing X [29]; (ii) paramagnetic honeycomb layers with alternating M(III) and M(I) ions [30]; and (iii) a chiral paramagnetic 3D network with alternating M(III) and M(I) ions [30]. All these heterometallic coordination polymers were prepared by using the SBU and complex-as-ligands approaches with different M(I) or M(II) ions and pre-formed [M^III^(C_6_O_4_X_2_)_3_]^3−^ building blocks [31]. In these systems, the open challenge is to achieve a control of the final structure and topology obtained since the 2D and 3D heterometallic networksare very close in energy, as evidenced by the simultaneous crystallization of both polymorphs in a single synthesis [30].

In order to rationalize the synthetic conditions leading to either 2D or 3D lattices, we explored different synthetic routes, changing the temperature, reagents ratios, presence of template molecules, and even the addition order. This study has led to the synthesis of a coordination polymer formulated as [(DAMS)_2_{FeNa(C_6_N_2_O_8_)_3_}·CH_3_CN]*_n_* (**1**) (DAMS^+^ = C_16_H_17_N_2_^+^ = 4-[4-(dimethylamino)-α-styryl]-1-methylpyridinium). This compound presents an original structure in an anilato-based compound. Interestingly, the network present in **1** has been observed in only two examples with the topologically related oxalato ligand [32,33]. In both cases, the anionic [Na^I^M^III^(C_2_O_4_)_3_]_2_ layers (M^III^ = Cr and Fe) are separated by layers with Na^+^ cations and water molecules or layers of the organic donor bis(ethylenedithio)tetrathiafulvalene (BEDT–TTF). This finding constitutes an additional proof that anilato and oxalato are very closely related ligands and that it is possible to extend all the chemistry performed with the oxalato ligand to the anilato-based ones.

## 2. Materials and Methods

All the reagents used were commercially available and were used as received without any further purification. The sodium salt of the nitranilate ligand, Na_2_[C_6_N_2_O_8_], was prepared as orange needles according to a method found in the literature [34].

### 2.1. Synthesis of the Precursor Salt Na_3_[Fe(C_6_N_2_O_8_)_3_]

A solution of FeCl_3_·6H_2_O (21.6 mg, 0.08 mmol) in H_2_O (2.5 mL) was added drop-wise to an aqueous solution (20 mL) of Na_2_[C_6_N_2_O_8_] (65.8 mg, 0.24 mmol). The resulting solution was heated at 60 °C to reduce the volume to 10 mL. The solution was cooled to obtain the precursor salt Na_3_[Fe(C_6_N_2_O_8_)_3_] as a deep orange crystalline powder (42.1 mg, yield 65%). Elemental Anal. Calc. for C_18_N_6_FeNa_3_O_24_ (*M*_w_ = 809.03): C, 26.72; N, 10.39. Found: C, 26.21; N, 10.18. FT-IR (ν_max_/cm^−1^, KBr pellet): 2962(m), 2934(m), 2874(m), 1624(m), 1560(s), 1396(s), 1316(m), 1270(w), 1099(w), 1047(m), 1022(m), 918(w), 861(m), 775(m), 571(w), 505(w).

### 2.2. Synthesis of [(DAMS)_2_{FeNa(C_6_N_2_O_8_)_3_}·CH_3_CN]_n_ (**1**)

A solution of the precursor salt Na_3_[Fe(C_6_N_2_O_8_)_3_] (8.09 mg, 0.01 mmol) and MnCl_2_.4H_2_O (1.98 mg, 0.01 mmol) in 4 mL of acetonitrile was mixed with a solution of 4-[4-dimethylamino)-α-styryl]-*N*-alkylpyridinium iodide (DAMSI) (3.66 mg, 0.01 mmol) in 4 mL of MeOH. The solution was left to evaporate at room temperature, resulting in the formation of prismatic red single crystals of **1** suitable for X-ray single crystal determination after four days. (4.48 mg, yield 35%). Elemental Anal. Calc. for C_52_H_38_FeN_11_NaO_24_ (*M*_w_ = 1279.75): C, 48.80; H, 2.99; N, 12.04. Found: C, 48.21; H, 3.18; N, 12.18. Electron probe microanalysis excluded the presence of Mn.

### 2.3. Single Crystal X-ray Structure Determination

A suitable single crystal of compound **1** was mounted on a glass fiber using a viscous hydrocarbon oil to coat the crystal and then transferred directly to the cold nitrogen stream for data collection. X-ray data were collected at 120 K on a Supernova Agilent Technologies diffractometer equipped with a graphite-monochromated Enhance (Mo) X-ray Source (λ = 0.71073 Å). The program CrysAlisPro, Agilent Technologies Ltd., was used for unit cell determinations and data reduction. Empirical absorption correction was performed using spherical harmonics, implemented in the SCALE3 ABSPACK scaling algorithm. Crystal structures were solved with direct methods with the SIR97 program [35], and refined against all F2 values with the SHELXL-2014 program [36], using the WinGX graphical user interface [37]. All non-hydrogen atoms were refined anisotropically, and hydrogen atoms were placed in calculated positions and refined isotropically with a riding model. There is a disorder in the CH_3_CN solvent molecules that appear with two possible orientations with a common N atom located on a C_2_ axis. Data collection and refinement parameters are given in Table 1.

CCDC-1457366 contains the supplementary crystallographic data for this paper. These data can be obtained free of charge from The Cambridge Crystallographic Data Center at www.ccdc.cam.ac.uk/data_request/cif.

### 2.4. Physical Measurements

IR spectra (400–4000 cm^−1^) were recorded with a Nexus Nicolet (Madison, WI, USA) FT-IR spectrophotometer in KBr pellets. Electron probe microanalysis was performed in a Philips SEM XL30 (Philips, Amsterdam, Netherland) equipped with an EDAX DX-4 microprobe.

Magnetic susceptibility measurements were carried out in the temperature range 2–300 K with an applied magnetic field of 0.1 T on a polycrystalline sample of compound **1** with an MPMS-XL-5 SQUID susceptometer (Quantum Design, San Diego, CA, USA). The susceptibility data were corrected for the sample holders previously measured using the same conditions and for the diamagnetic contribution of the salt as deduced by using Pascal’s constant tables (χ_dia_ = −619.1 × 10^−6^) [38].

## 3. Results

### 3.1. Synthesis of Compound [(DAMS)_2_{FeNa(C_6_N_2_O_8_)_3_}·CH_3_CN]_n_ (**1**)

The synthesis of the title compound was performed using equimolar amounts of the pre-formed complex [Fe(C_6_N_2_O_8_)_3_]^3−^, prepared as its Na^+^ salt, Mn(NO_3_)_2_·4H_2_O, and the cation DAMS^+^ (=4-[4-(dimethylamino)-α-styryl]-*N*-alkylpyridinium). It is interesting to note that the Mn(II) ions do not appear in the final product, but play an important role in the synthesis since all the attempts to prepare compound **1** without the addition of Mn(II) ions failed. Given the strong affinity of Mn(II) for the oxygen-containing ligands [39], we presume that the Mn(II) ions may help to the formation of this original structure by the initial coordination to the NO_2_ groups of different [Fe(C_6_N_2_O_8_)_3_]^3−^ complexes. In this way, the Fe(III) complexes get close, as observed in the structure. The Na^+^ cations present in the structure come from the precursor salt of the [Fe(C_6_N_2_O_8_)_3_]^3−^ complex.

### 3.2. Crystal Structure of Compound [(DAMS)_2_{FeNa(C_6_N_2_O_8_)_3_}·CH_3_CN]_n_ (**1**)

**Figure 1:**
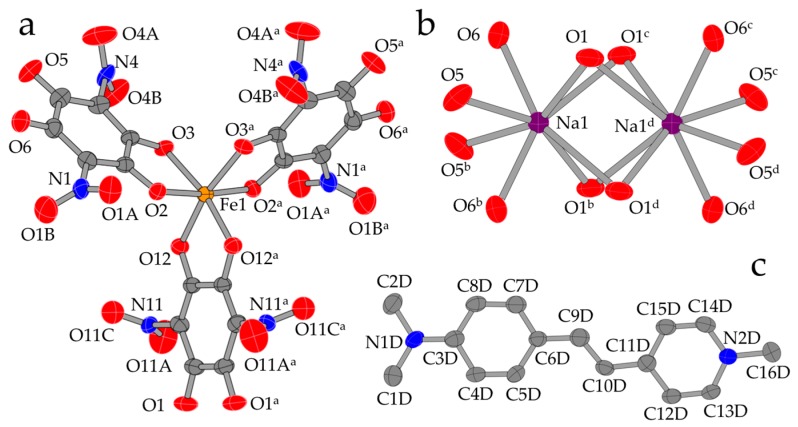
Ortep views of the fragments of the structure of compound **1** with the labeling scheme: (**a**) structure of the [Fe(C_6_N_2_O_8_)_3_]^3−^ unit; (**b**) structure of the Na_2_O_12_ dimer; (**c**) structure of the DAMS^+^ cation.

The asymmetric unit of compound **1** contains one [Fe(C_6_N_2_O_8_)_3_]^3−^ unit (Figure 1a), located in a C_2_ axis, one Na^+^ cation located on a C_2_ axis (Figure 1b), and one DAMS cation located on a general position (DAMS^+^ = 4-[4-(dimethylamino)-α-styryl]-*N*-alkylpyridinium) (Figure 1c).

**Figure 2:**
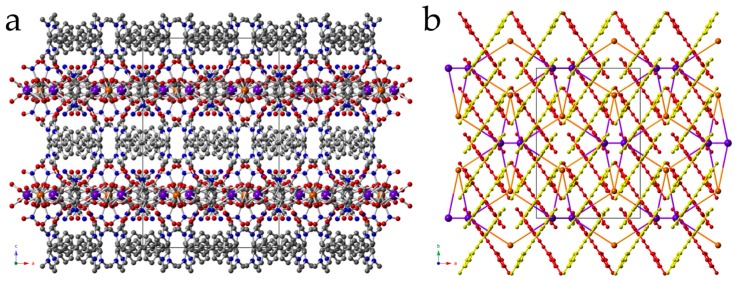
(**a**) View of the alternating cationic and anionic layers in compound **1**. H atoms have been omitted for clarity; (**b**) View along the *c* direction of two consecutive cationic layers showing the different orientation of the DAMS^+^ molecules in each layer (yellow and red). The anionic intermediate layer is only represented by the Fe(III) and Na^+^ ions (orange and purple, respectively).

**Figure 3:**
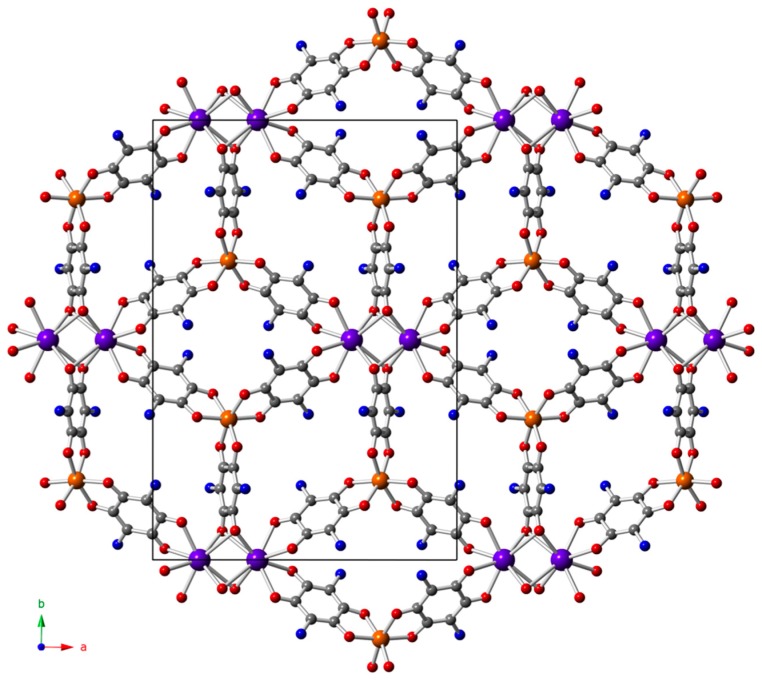
View of the 3,6-connected anionic layer [NaFe(C_6_N_2_O_8_)_3_]^2−^ generated with Fe(III) and pairs of Na^+^ cations (the oxygen atoms of the NO_2_ groups have been omitted for clarity). Color code: Fe = orange, Na = purple, O = red, N = blue and C = grey.

The structure of compound **1** is formed by cationic and anionic layers parallel to the *ab* plane alternating along the *c* direction (Figure 2). The anionic layers can be formulated as [Na_2_Fe_2_(C_6_N_2_O_8_)_6_]^4−^ and are formed by [Fe(C_6_N_2_O_8_)_3_]^3−^ anions and Na^+^ cations. The structure of these layers can be described as a 3,6-connected 2D coordination polymer derived from the well-known hexagonal honeycomb lattice with Fe(III) and pairs of Na^+^ cations located in alternating vertices and [C_6_N_2_O_8_]^2−^ ligands forming the sides of the hexagons (Figure 3). There are, albeit, two important differences: (i) In **1**, the vertices of the hexagons contain dimers of Na^+^ cations where the Na^+^ cations are connected through four oxygen atoms from two [C_6_N_2_O_8_]^2−^ ligands (Figure 1b); and (ii) there is an additional [Fe(C_6_N_2_O_8_)_3_]^3−^ anion in the center of the hexagons with the three nitranilate ligands pointing towards the Na^+^ pairs, (Figure 3), giving rise to a final lattice that can be formulated as [Na_2_Fe_2_(C_6_N_2_O_8_)_6_]^4−^ with the Schläfli symbol (4^3^)_2_(4^6^.6^6^.8^3^). The four negative charges are balanced by four DAMS^+^ cations located between the anionic layers (Figure 2). There is one disordered acetonitrile solvent molecule located in the anionic layer.

The Fe(III) ions are surrounded by three bis-bidentate [C_6_N_2_O_8_]^2−^ anions that connect each Fe(III) with a pair of Na^+^ cations and two Na^+^ cations from two different Na_2_^2+^ pairs (Figure 3). The coordination around the Fe(III) ions is a distorted octahedron with Fe–O bond lengths in the range 1.997–2.018 Å (Table 2), similar to those found in other related [Fe(C_6_O_4_X_2_)_3_]^3−^ complexes [29,30,31].

The NO_2_ groups of the ligands are tilted with respect to the anilato ring angles in the range 48.9°–77.5°, as observed in other compounds containing the nitranilate ligand [31,40]. The Na^+^ cation is located on a C_2_ axis and close to a second C_2_ axis perpendicular to one containing the Na^+^ cation. This second C_2_ axis generates pairs of Na^+^ cations connected through four O1 oxygen atoms (O1, O1^b^, O1^c^, and O1^d^, Figure 1b). Each Na^+^ cation appears surrounded by a total of eight oxygen atoms: O5, O5^b^, O6, and O6^b^ plus the four bridging O1 atoms (Figure 1b). The coordination geometry around the Na^+^ cations can be defined as a distorted triangular dodecahedron with six short Na–O bond distances (in the range 2.419–2.467 Å) and two long ones (2.879(4) Å). The Na–Na^d^ distance through the quadruple oxido bridge is 3.256(4) Å (Table 2).

The organic layers are formed by one independent DAMS^+^ cation. The presence of an inversion center near the DAMS^+^ cations generates pairs of parallel DAMS^+^ cations with opposite orientations of the dimethylamino groups (Figure 2b). The DAMS^+^ dimers are packed in stacks running along the *b* direction (Figure 2b). In each layer, the DAMS^+^ cations are parallel and form an angle of 70.8° with the DAMS^+^ cations of the neighboring layers (Figure 2b).

There are no interlayer interactions worth mentioning since the shortest O···H interlayer distance between the oxygen atoms of the ligand and the H atoms of the cation is above 2.4 Å.

### 3.3. Magnetic Properties of Compound [(DAMS)_2_{FeNa(C_6_N_2_O_8_)_3_}·CH_3_CN]_n_ (**1**)

**Figure 4:**
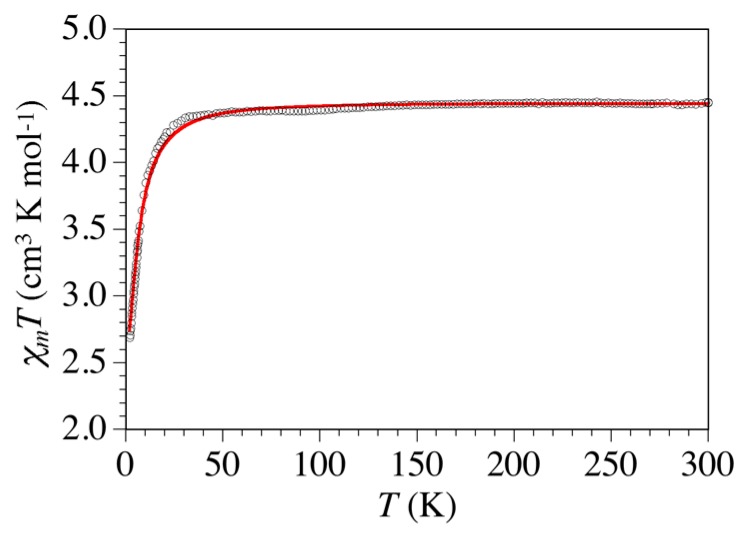
Thermal variation of the χ_m_*T* product per Fe(III) ion for compound **1**. Solid line is the best fit to the model (see text).

The product of the molar magnetic susceptibility times the temperature of compound **1** per Fe(III) ion shows, at room temperature, a value of *ca.* 4.5 cm^3^·K·mol^−1^, close to the expected one for a high spin S = 5/2 Fe(III) ion (Figure 4). When the sample is cooled, χ_m_*T* remains constant down to *ca.* 20 K. Below this temperature, χ_m_*T* shows an abrupt decrease to reach a value of *ca.* 2.7 cm^3^·K·mol^−1^ at 2 K. Since the Fe(III) centers are quite well isolated by the Na^+^ dimers, this abrupt decrease has to be attributed to the presence of a zero field splitting in the Fe(III) ions (see below).

## 4. Discussion

The structure of compound **1** is quite original since it has never been observed in any anilato-based compound; as far as we know, it has only been obtained in two examples with the oxalato ligand, both containing the organic donor bis(ethylendithio)tetrathiafulvalene (BEDT–TTF) [32,33]. Although, as mentioned above, there are no short interlayer interactions, it is interesting to note that both molecules (BEDT–TTF and DAMS) are very similar in size and geometry. In our case, the formation of this original structure seems to be facilitated by the presence of Mn(II) ions and, most importantly, of the Na^+^ cations. This assumption is based on the fact that, when using the same synthetic conditions with the DMAS^+^ cations and the oxalato ligand (except for the presence of Na^+^ cations), the obtained structure is the usual honey comb [MnCr(C_2_O_4_)_3_]^−^ lattice [41].

An additional original aspect of this structure is the presence of a Na^+^ dimer with a bridge formed by four oxygen atoms (Figure 1b). In fact, a search in the CSD database (updated to Nov. 2015) [42] shows only 19 of such NaO_4_Na dimeric units, including three NaO_4_NaO_4_Na trimers [43,44,45]. In these NaO_4_Na units, the oxygen bridges belong to different coordinating groups as carboximidato (R-C=NO–Na, in five cases) [46,47,48], hydroxamato (H-C=NO–Na, in three cases) [43,49,50], water molecules (in three cases) [51,52,53], acetato (in two cases) [44,45], oxalato (in two cases) [54], ketone (in two cases) [55,56], alkoxido (in one case) [57], and one more case with two H_2_O molecules and two NO_2_ groups [58]. Compound **1** is the first example where the four bridging oxygen atoms belong to an anilato group.

The Na–Na distances in these 19 examples range from 2.857 to 4.042 Å with an average value of 3.267 Å, very close to the one observed in **1** (3.256(4) Å).

Compound **1** possesses an inversion center and, therefore, is not expected to show any non-linear optical (NLO) response, despite containing the DAMS^+^ cation, which is well-known to provide large second-order NLO responses [41,59].

The magnetic properties of compound **1** are the expected ones for isolated high spin *S* = 5/2 Fe(III) ions since the Na^+^ dimers preclude any exchange interaction between the Fe(III) ions. This situation is very similar to that observed in other anilato-based 2D and 3D structures with paramagnetic Fe(III) or Cr(III) centers separated by Na^+^ or K^+^ cations [30]. Accordingly, we have fit the magnetic properties to a simple model for an *S* = 5/2 monomer with a zero field splitting [60] accounting for the sharp decrease of χ_m_*T* at low temperatures. This simple model reproduces very satisfactorily the magnetic properties of compound **1** with *g* = 2.016 and |*D*| = 8.5 cm^−1^ (solid line in Figure 4). This value is similar to those found in other Fe(III) complexes [61] and may include a weak antiferromagnetic interaction between the Fe(III) centers. Note that the sign of *D* cannot be determined from powder susceptibility measurements.

## 5. Conclusions

The use of the nitranilate ligand with Fe(III) and Na^+^ ions led to a coordination polymer with an unprecedented 3,6-connected 2D structure derived from the well-known honey comb 2D hexagonal lattice with two important differences: (i) the vertices of the hexagons were occupied by Na^+^ dimers alternating with Fe(III) centers; and (ii) there was an additional [Fe(C_6_N_2_O_8_)_3_]^3−^ complex occupying the center of the hexagons connecting the three pairs of Na^+^ ions. This original arrangement resulted in an anionic lattice that can be formulated as [Na_2_Fe_2_(C_6_N_2_O_8_)_6_]^4−^, whose charge is neutralized by four DAMS^+^ cations. This compound represents an additional proof that the anilato derivative ligands are topologically similar to the oxalato with the advantage that anilato derivatives can be easily functionalized.

## Figures and Tables

**Table 1:** Crystal data and structure refinement of compound **1**.

Compound	1
Formula	C_52_H_41_FeN_11_NaO_24_
F. *W*_t_.	1,282.80
Crystal system	Orthorhombic
Space group	Ccca
*a* (Å)	17.0607(8)
*b* (Å)	24.6580(12)
*c* (Å)	26.2191(14)
α (°)	90
β (°)	90
γ (°)	90
*V* (Å^3^)	11,029.9(10)
*Z*	8
*T* (K)	120
$\rho_{\mathrm{calc}}$ (g.cm^−3^)	1.545
μ (cm^−1^)	0.379
F(000)	5272
Crystal size (mm^3^)	0.12 × 0.09 × 0.05
θ range (°)	2.86–25.06
Total reflections	39,529
Unique reflections	4,885
*R*_int_	0.1194
Data with *I* > 2σ(I)	2,904
*N*_v_	425
^a^ *R*1	0.0613
^b^ w*R*2	0.1214
^c^ GooF	1.057
Δ$\rho_{\max,\;\min}$ (eÅ^−3^)	+0.626
Δ$\rho_{\max,\;\min}$ (eÅ^−3^)	−0.386

^a^ *R*1 = Σ|*F*_o_ − *F*_c_|/*F*_o_; ^b^ w*R*2 = {Σ[w(*F*_o_^2^ − *F*_c_^2^)^2^]/Σ[w(*F*_o_^2^)^2^]}^1/2^; ^c^ GooF = {Σ[w(*F*_o_^2^ − *F*_c_^2^)^2^]/(*N*_obs_ − *N*_var_)}^1/2^.

**Table 2:** Main bond lengths (Å) and angles (°) in compound **1**.

**Atoms**	**Distance**	**Atoms**	**Distance**
Fe1–O2	1.995(2)	Na1–O1 ^c^	2.876(3)
Fe1–O2 ^a^	1.995(2)	Na1–O1 ^d^	2.876(3)
Fe1–O3	2.016(2)	Na1–O5	2.466(3)
Fe1–O3 ^a^	2.016(2)	Na1–O5 ^b^	2.466(3)
Fe1–O12	2.016(2)	Na1–O6	2.417(3)
Fe1–O12 ^a^	2.016(2)	Na1–O6 ^b^	2.417(3)
Na1–O1	2.428(3)	Na1–Na1 ^d^	3.256(4)
Na1–O1 ^b^	2.428(3)		
**Atoms**	**Angle**	**Atoms**	**Angle**
O2–Fe1–O2 ^a^	172.77(14)	O3–Fe1–O12	166.86(10)
O2–Fe1–O3	79.74(10)	O3 ^a^–Fe1–O12	95.48(10)
O2 ^a^–Fe1–O3	95.18(10)	O2–Fe1–O12 ^a^	96.89(10)
O2–Fe1–O3 ^a^	95.18(10)	O2 ^a^–Fe1–O12 ^a^	88.67(10)
O2 ^a^–Fe1–O3 ^a^	79.71(10)	O3–Fe1–O12 ^a^	95.48(10)
O3–Fe1–O3 ^a^	91.75(15)	O3 ^a^–Fe1–O12 ^a^	166.86(10)
O2–Fe1–O12	88.67(10)	O12–Fe1–O12 ^a^	79.66(15)
O2 ^a^–Fe1–O12	96.89(10)		

Symmetry operations: a = −x, y, −z + 1/2; b = x, −y + 1/2, −z + 1/2; c = −x − 1, y, −z + 1/2; d = −x − 1, −y + 1/2, z.
